# Neurophysiological Differentiation of Conversion to Alzheimer's Disease: Longitudinal Changes in P300 Amplitude as an Indicator of Mild Cognitive Impairment Progression

**DOI:** 10.1002/brb3.71555

**Published:** 2026-07-08

**Authors:** Yağmur Özbek, Görsev G. Yener

**Affiliations:** ^1^ Faculty of Medicine Izmir University of Economics Izmir Türkiye; ^2^ Department of Neuroscience Institute of Health Sciences, Dokuz Eylül University Izmir Türkiye; ^3^ Faculty of Medicine, Department of Neurology Dokuz Eylül University Izmir Türkiye

**Keywords:** Alzheimer's disease conversion, mild cognitive impairment, P300, longitudinal

## Abstract

**Purpose:**

This longitudinal study investigated whether P300 event‐related potential (ERP) amplitude demonstrates group‐level and longitudinal differences between stable and progressive mild cognitive impairment (MCI), and whether these electrophysiological changes may serve as preliminary markers associated with Alzheimer's disease (AD) progression.

**Method:**

Fifty‐four participants, including 27 cognitively unimpaired (CU) individuals and 27 individuals with multi‐domain amnestic MCI, underwent EEG recording during a visual oddball task at baseline and follow‐up assessment. Within 1 year, 14 MCI participants progressed to AD (pMCI), while 13 remained stable (sMCI). P300 amplitudes were compared across groups and over time, and exploratory receiver operating characteristic (ROC) analyses were conducted to evaluate discriminative performance. Correlations between P300 amplitudes and cognitive measures were also examined.

**Findings:**

Baseline analyses demonstrated significantly reduced P300 amplitudes in both MCI groups compared to CU participants (*p* < 0.001), consistent with early cortical dysfunction. Longitudinally, CU and sMCI groups exhibited amplitude decline over time, whereas the pMCI group showed persistently stable but markedly low amplitudes. Exploratory ROC analyses suggested potential group‐level discriminative utility for differentiating pMCI from both CU and sMCI participants. In addition, lower P300 amplitudes were significantly associated with poorer global cognitive performance and memory scores.

**Conclusion:**

These findings suggest that reduced P300 amplitude, particularly at central electrode locations, may represent a preliminary electrophysiological marker associated with progression from MCI to AD. The results support the potential relevance of P300 measures in future prognostic and longitudinal biomarker research in Alzheimer's disease.

## Graphical abstract

Longitudinal EEG recordings showed reduced P300 amplitudes in mild cognitive impairment, with the lowest values observed in individuals who progressed to Alzheimer's disease. Central P300 measures demonstrated strong discriminative performance and may represent an accessible electrophysiological marker associated with disease progression.

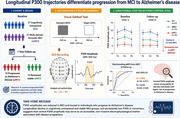

## 1 Introduction

Alzheimer's Disease (AD) is the most common cause of dementia, accounting for approximately 60%–80% of all cases and affecting approximately 57 million individuals worldwide (WHO 2025). In response to recent advances in AD diagnostics and therapeutics, the Alzheimer's Association updated its 2018 research framework to define AD as a biological continuum independent of clinical symptomatology. Central to this revision is the emphasis on early‐changing core biomarkers such as amyloid PET, cerebrospinal fluid (CSF) measures, and plasma phosphorylated tau as sufficient for establishing an AD diagnosis even in asymptomatic individuals (Jack Jr et al. 2024). These developments have intensified interest in identifying additional accessible and non‐invasive markers that may help characterize early disease‐related changes. Tanaka et al. 2024.

Mild Cognitive Impairment (MCI) represents an intermediate stage between expected age‐related cognitive decline and dementia, characterized by measurable deficits in one or more cognitive domains that do not substantially impair independence in daily functioning. MCI is clinically heterogeneous and may include amnestic or non‐amnestic forms, as well as single‐domain or multidomain presentations. The present study focuses specifically on multidomain amnestic MCI, a subtype associated with elevated risk for progression to AD (Oltra‐Cucarella et al. 2018). However, prognosis remains variable. While some individuals progress to dementia, others remain cognitively stable or, less commonly, revert to normal cognition over time. Annual conversion rates from MCI to dementia have been estimated at approximately 10%–15% in clinical cohorts, while population‐based studies report lower rates around 6%–10% (Petersen et al. 2018; Salemme et al. 2025). It has been suggested that conversion risk is highest within the first 3 years and decreases with longer follow‐up, indicating earlier progression among higher‐risk cases (Salemme et al. 2025). These findings highlight the heterogeneous prognosis of MCI. Factors influencing progression include MCI subtype, presence of AD biomarkers, APOE ε4 genotype, and vascular or psychiatric comorbidities (Albert et al. 2011; Jack et al. 2018). Importantly, individuals with biomarker‐confirmed MCI due to Alzheimer's pathology may exhibit substantially higher progression rates (Dubois et al. 2021; Öksüz et al. 2024).

Event‐related potentials (ERPs), recorded using electroencephalography (EEG), provide non‐invasive and temporally precise measures of cognitive processing during task performance. Among ERP components, the P300, commonly elicited using auditory or visual oddball paradigms, is thought to index attentional resource allocation and working memory updating (Polich 2007). Numerous cross‐sectional studies have demonstrated reduced P300 amplitude and prolonged latency in individuals with MCI and AD compared with cognitively unimpaired (CU) adults, reflecting impairments in attention and working memory processes (Yamaguchi et al. 2000; Bennys et al. 2007; Demirayak et al. 2023; Liang et al. 2025; Tanaka et al. 2024).

Although both amplitude and latency abnormalities have been reported, the present study prioritized P300 amplitude as the primary endpoint because prior evidence suggests it may be particularly sensitive to progression‐related differences and alterations in cognitive resource allocation (Polich 2007; Demirayak et al. 2023). Findings regarding the ability of P300 measures to distinguish stable from progressive MCI, however, remain inconsistent. Some studies have suggested that prolonged baseline P300 latency may be associated with increased risk of progression (Papaliagkas et al. 2008; Vecchio and Määttä 2014), whereas others have reported limited discriminatory value for distinguishing progressive from stable MCI (Olichney et al. 2008; Vecchio and Määttä 2014). Thus, the potential role of P300 as a marker differentiating MCI trajectories remains unresolved.

Longitudinal studies may help clarify whether P300 alterations reflect stable features of impairment or dynamic changes associated with progression. However, relatively few electrophysiological studies have followed individuals with MCI over time, and even fewer have explicitly differentiated stable MCI (sMCI) from progressive MCI (pMCI) using longitudinal ERP trajectories (Yener and Başar 2010; Papaliagkas et al. 2008; Yener et al. 2016). This gap is important, as it remains unclear whether baseline P300 abnormalities or their evolution over time are systematically associated with subsequent progression to AD (Olichney et al. 2008; Vecchio and Määttä 2014).

Because P300 exhibits a distributed fronto‐central‐parietal topography, examining electrode‐specific responses may provide additional information regarding progression‐related neurophysiological changes. Frontal (*F_z_*), central (*C_z_*), and parietal (*P_z_*) sites may reflect partially distinct contributions of executive control, attentional allocation, and working memory processes, and may differ in their sensitivity to pathological cognitive aging (Polich 2007; Yener and Başar 2010). Examining multiple electrode locations may therefore improve characterization of progression‐related effects.

In addition, associations between P300 measures and neuropsychological performance may provide convergent evidence regarding the cognitive relevance of electrophysiological changes. Neuropsychological measures assessing global cognition, episodic memory, attention, executive function, and semantic retrieval are sensitive to deficits commonly observed in MCI and may help contextualize the functional significance of ERP abnormalities.

Neuropsychological assessment plays a central role in the characterization of cognitive impairment in MCI and provides important information regarding the functional significance of neurophysiological alterations (Albert et al. 2011; Petersen et al. 2018). Commonly used cognitive measures assess domains frequently affected during prodromal AD, including episodic memory, executive functioning, attention, language, and semantic retrieval (Salmon and Bondi 2009; Gauthier et al. 2006). Verbal fluency tasks, for example, are considered sensitive indicators of executive and semantic network dysfunction and are commonly impaired in individuals with progressive cognitive decline (Henry et al. 2004; Clark et al. 2009). Similarly, global cognitive screening measures such as the Mini‐Mental State Examination (MMSE) provide an overall estimate of cognitive functioning (Folstein et al. 1975), while memory‐oriented tests are particularly relevant given the prominent episodic memory deficits observed in amnestic MCI and early AD (Dubois et al. 2007; Petersen et al. 2014). Associations between ERP measures and neuropsychological performance may therefore help clarify the cognitive processes reflected by P300 alterations and provide insight into the relationship between electrophysiological dysfunction and clinical symptom progression (Polich 2007; Bennys et al. 2007).

Accordingly, the primary aim of this study was to evaluate whether baseline P300 amplitudes differ among cognitively unimpaired individuals, stable MCI (sMCI), and progressive MCI (pMCI), and whether these groups show distinct longitudinal trajectories over 1 year. A secondary aim was to examine whether electrode‐specific P300 amplitudes recorded at *F_z_*, *C_z_*, and *P_z_* provide differential sensitivity to progression‐related changes. Finally, we explored associations between P300 measures and neuropsychological performance to provide preliminary evidence regarding the potential relevance of these electrophysiological markers for future prognostic biomarker research.

## 2 Materials and Methods

### 2.1 Participants

**FIGURE 1:**
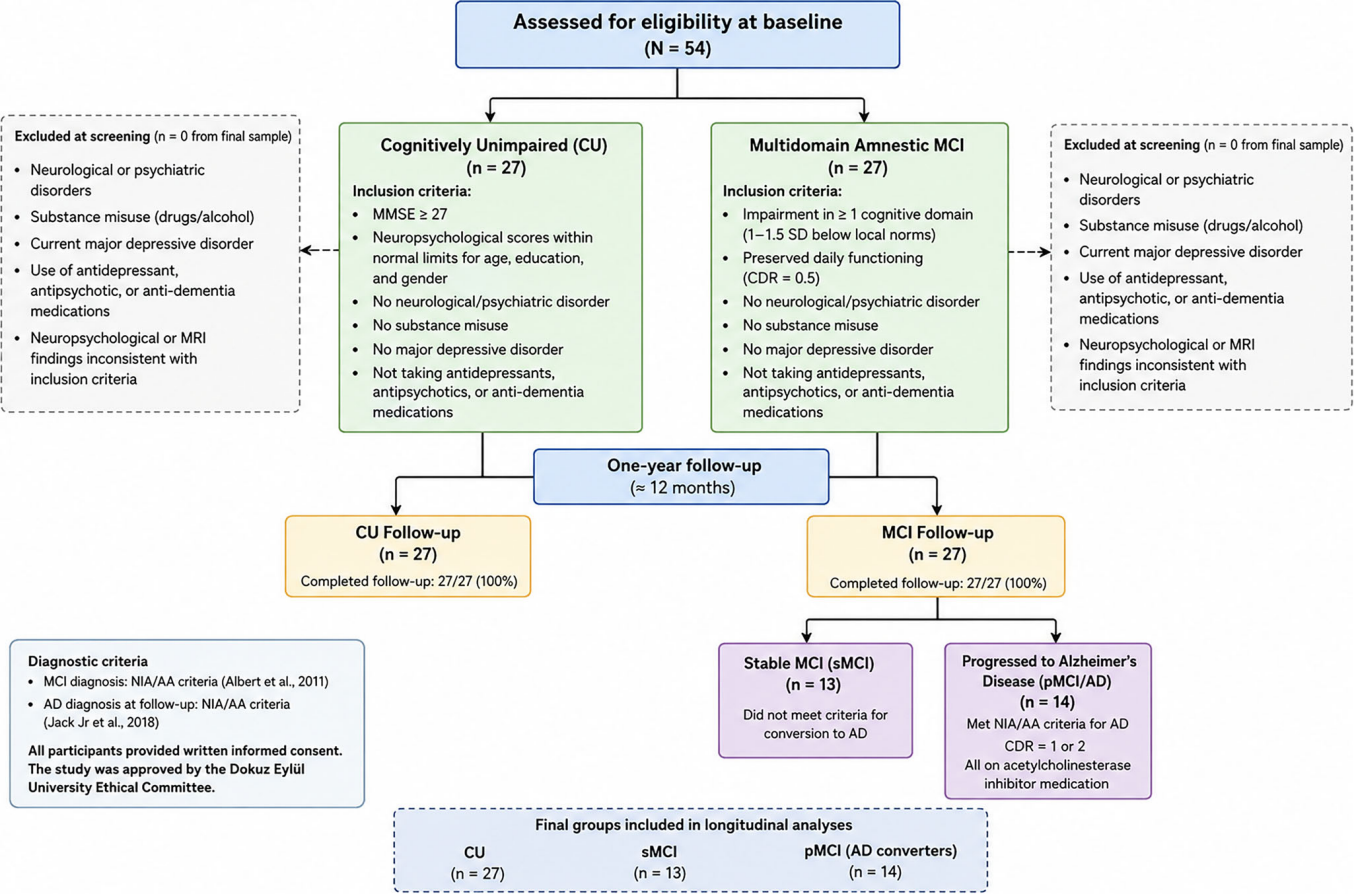
Recruitment flow of participants.

**FIGURE 2:**
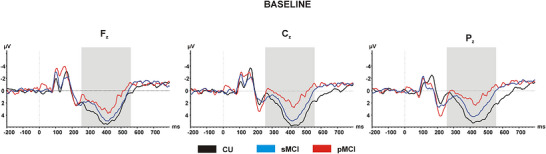
Baseline P300 mean amplitudes across groups. The grey shaded region indicates the P300 measurement window (250–550 ms). At baseline, the Mild Cognitive Impairment stable subgroup (sMCI) and progressive subgroup (pMCI) showed lower P300 amplitudes than cognitively unimpaired (CU) individuals, with sMCI exhibiting intermediate amplitudes between CU and pMCI. pMCI showed the lowest amplitudes across groups.

**FIGURE 3:**
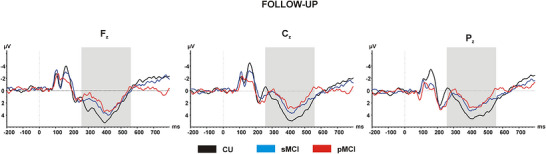
Follow‐up P300 mean amplitudes across groups. The grey shaded region indicates the P300 measurement window (250–550 ms). At follow‐up no significant difference between sMCI and pMCI groups while CU showed higher amplitudes than both sMCI and pMCI.

**FIGURE 4:**
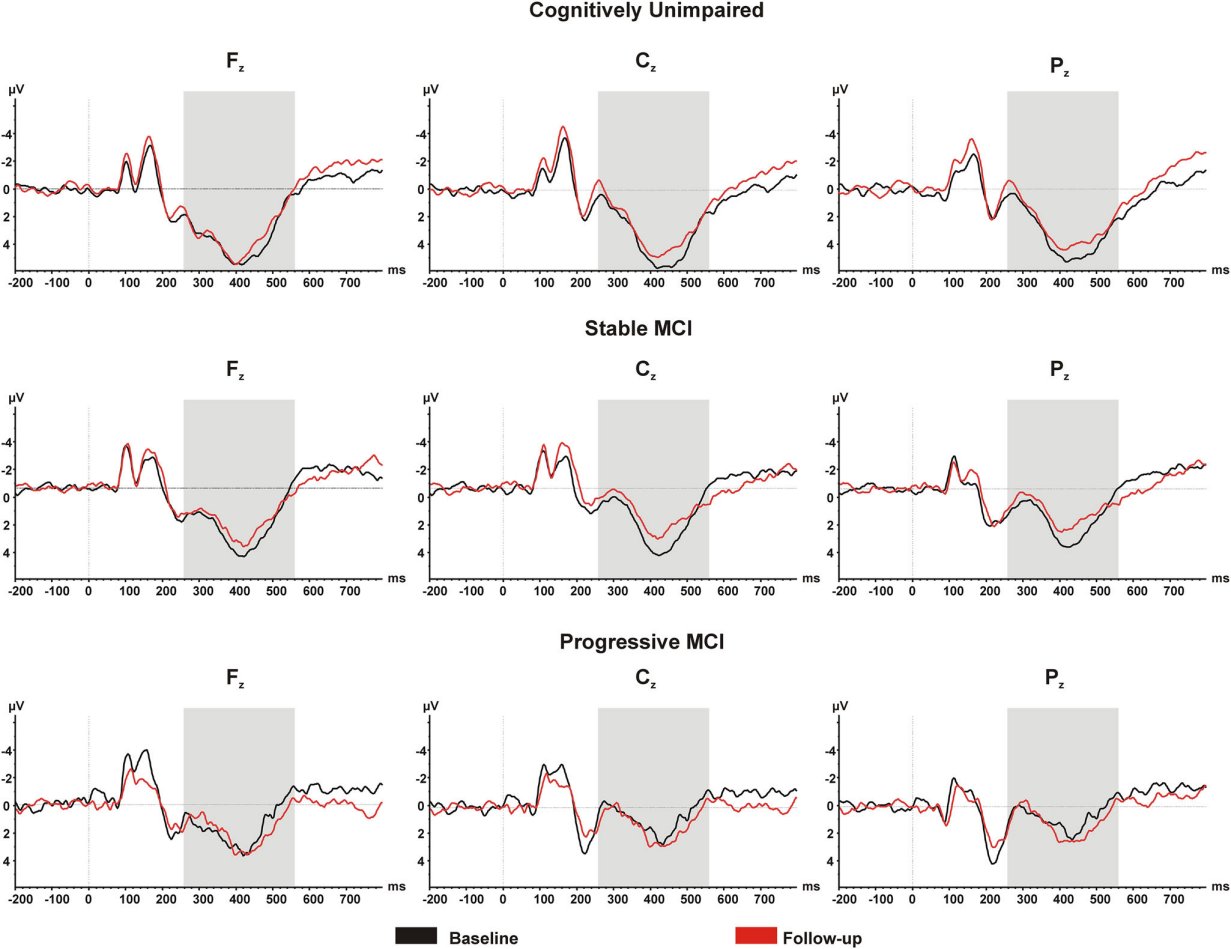
Baseline and follow‐up P300 mean amplitudes within groups. The grey shaded region indicates the P300 measurement window (250–550 ms). CU and sMCI had higher P300 amplitudes at baseline compared to follow‐up.

**FIGURE 5:**
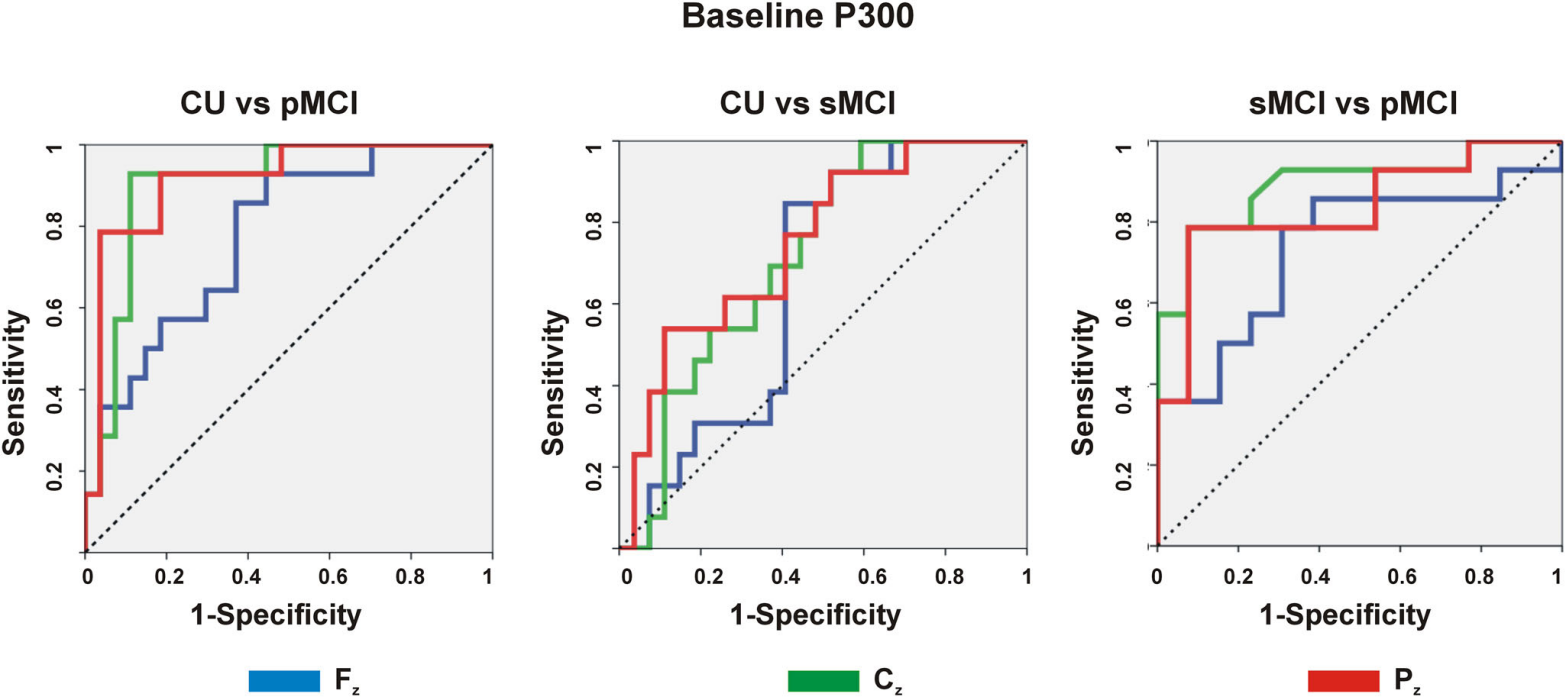
ROC curves of baseline P300 amplitudes across CU and sMCI, CU and pMCI, and sMCI and pMCI at *F_z_*, *C_z_*, and *P_z_* electrodes.

**FIGURE 6:**
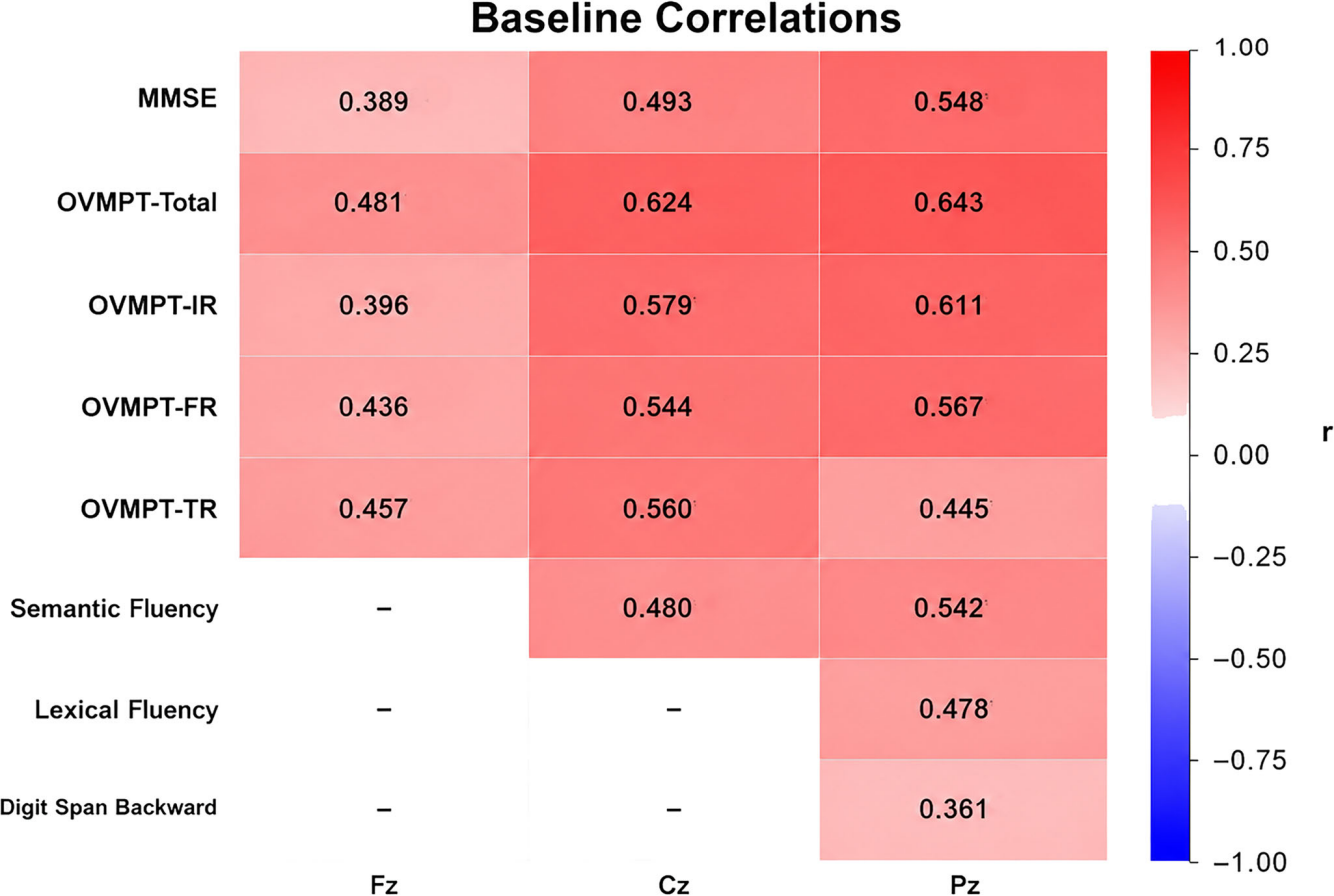
Baseline correlations between neuropsychological test scores and P300 amplitudes.

**FIGURE 7:**
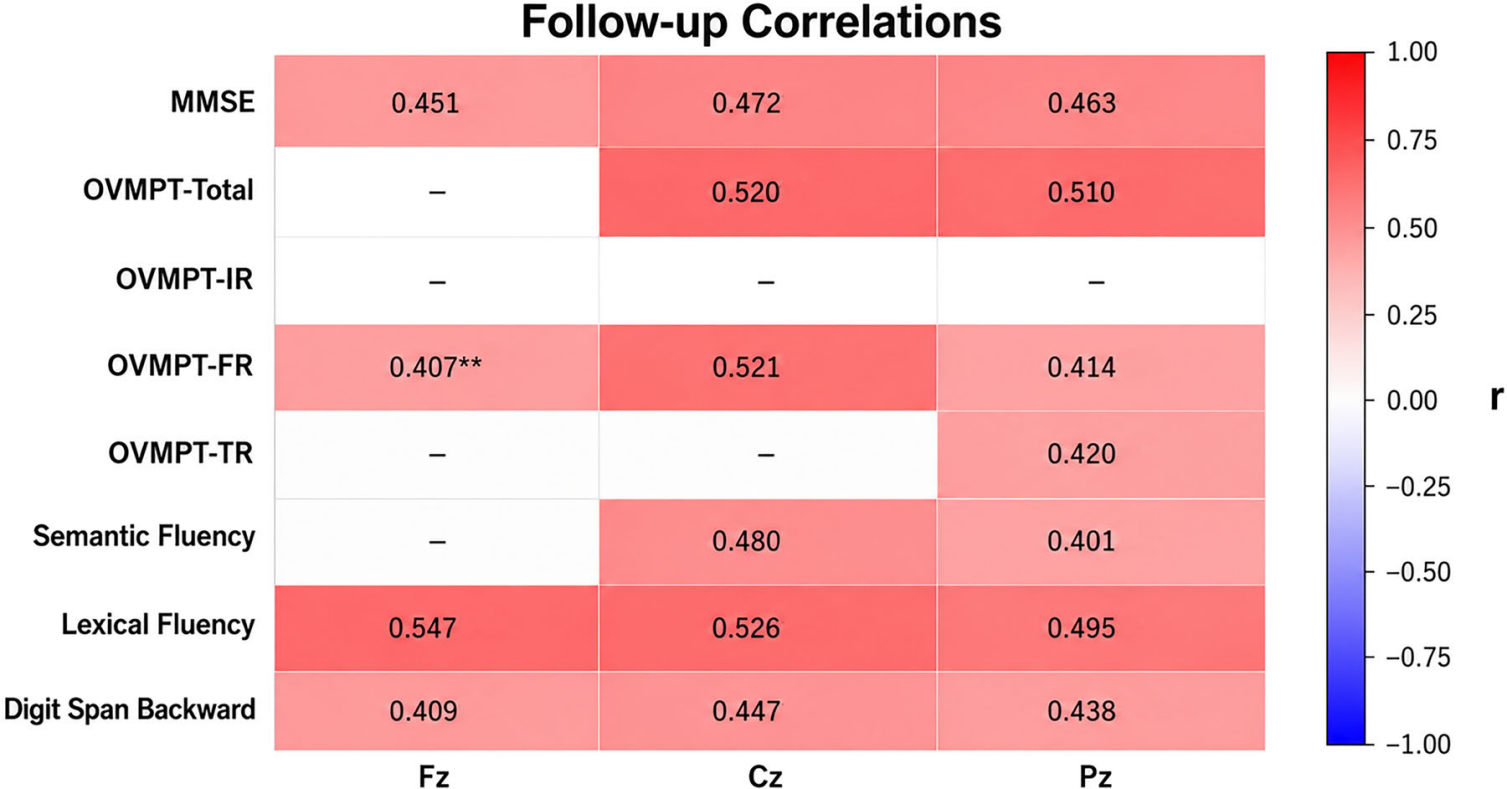
Follow‐up correlations between neuropsychological test scores and P300 amplitudes.

The study included 27 cognitively unimpaired individuals (CU) and 27 individuals with multi‐domain amnestic MCI. The MCI diagnosis was made by a neurologist following the NIA/AA criteria (Albert et al. 2011). All the participants underwent a comprehensive neuropsychological evaluation and magnetic resonance imaging (MRI) for baseline and follow‐up visits after 1 year. At the baseline inclusion criteria for CU are as follows: (1) participants must not have a history or current diagnosis of neurological or psychiatric disorders; (2) participants must have no history of substance misuse, including drugs and alcohol; (3) participants must achieve a minimum score of 27 on the Mini‐Mental State Examination; (4) neuropsychological test scores must align with local norms adjusted for age, education, and gender; (5) participants must not have a current diagnosis of major depressive disorder; and (6) participants must not be taking antidepressants, anti‐dementia, or antipsychotic medications. At the baseline inclusion criteria for MCI are as follows: (1) participants must not have any history or current presence of other neurological or psychiatric conditions; (2) participants must not have a history of drug or alcohol misuse; (3) participants must show impairment in one or more cognitive domains, as evidenced by neuropsychological test scores falling 1–1.5 standard deviations below local norms; (4) participants must maintain functionality in their daily living activities, as indicated by a Clinical Dementia Rating Scale (CDR) score of 0.5; (5) participants must not have a current diagnosis of MDD); and (6) participants must not be taking antidepressants, anti‐dementia, or antipsychotic medications. All participants followed up approximately 1 year after baseline. A diagnosis of AD and MCI was made by a neurologist by following the NIA/AA criteria (Jack et al. 2018). It was seen that 14 of the 27 individuals with MCI convert to AD. Inclusion criteria for AD is as follows: (1) Participants must not have a history of drug or alcohol misuse; (2) participants must have impaired functionality in their daily living activities, as indicated by a Clinical Dementia Rating Scale (CDR) score of one or two; and (3) participants must not have a current diagnosis of MDD. All individuals with AD were on acetylcholinesterase inhibitor medications during experimental procedures in follow‐up (Figures 1, 2, 3, 4, 5, 6, 7).

All participants provided written informed consent according to the Helsinki Declaration. This study was approved by the Dokuz Eylül University Ethical Committee.

The clinical and demographic information of participants are presented in Table 1.

**TABLE 1:** Demographic and clinical characteristics of participants.

	CU (*n* = 27)	sMCI (*n* = 13)	pMCI (*n* = 14)	*p*
**Age (years)^a^**	72.67 ± 5.79	75.46 ± 5.77	75.93 ± 4.81	0.140
**Education (years)^a^**	10.85 ± 5.56	9.31 ± 4.25	7.57 ± 3.87	0.131
**Gender (M/F)^b^**	10/17	7/6	6/8	0.459
**GDS—baseline^a^**	6.96 ± 5.50	10.23 ± 7.22	8.71 ± 5.06	0.252
**GDS—follow‐up^a^**	6.68 ± 5.27	9.31 ± 7.67	7.14 ± 4.98	0.418
**Epoch number—baseline^a^**	29.59 ± 5.49	29.39 ± 7.12	28.98 ± 5.97	0.533
**Epoch number—follow‐up^a^**	29.59 ± 5.32	28.77 ± 7.20	26.50 ± 5.86	0.279

CU: cognitively unimpaired, sMCI: stable mild cognitive impairment, pMCI: progressive mild cognitive impairment, M: male, F: female, GDS: Geriatric Depression Scale.

^a^One‐way ANOVA.

^b^Chi‐square test.

### 2.2 Neuropsychological Testing

All participants underwent a detailed neuropsychological testing twice first at the baseline and approximately 1 year after baseline evaluation in the follow‐up. The neuropsychological battery included Mini Mental State Examination, Oktem Verbal Memory Processes Test (OVMPT), Stroop Test, semantic and lexical fluency tests, Boston Naming Test‐15 item (BNT‐15), digit span forward and digit span backward. The analysis of OVMPT included four sub‐scores from OVMPT as total score, immediate recall, delayed recall and total recognition. The neuropsychological tests are applied by a neuropsychologist. In order to eliminate learning effect, different forms of tests are used in baseline and follow‐up when it is applicable. The neuropsychological test results of participants are presented in Tables 2 and 3.

**TABLE 2:** Baseline neuropsychological test scores of participants.

	CU (*n* = 27)	sMCI (*n* = 13)	pMCI (*n* = 14)	*p*
**MMSE^a,b^**	28.80 ± 1.17	26.08 ± 3.30	23.86 ± 3.61	**<0.001**
**OVMPT‐Total score^a,b^**	114.41 ± 10.67	73.15 ± 13.76	70.43 ± 17.16	**<0.001**
**OVMPT‐IR^a,b^**	5.52 ± 1.25	3.69 ± 1.25	3.50 ± 1.74	**<0.001**
**OVMPT‐FR^a,b^**	13.15 ± 1.35	6.62 ± 4.09	5.71 ± 3.02	**<0.001**
**OVMPT‐TR^a^**	14.96 ± 0.20	14.08 ± 1.55	12.71 ± 1.64	**<0.001**
**Stroop interference (sec)**	52.00 ± 18.00	65.38 ± 35.77	68.64 ± 46.54	0.266
**Lexical fluency^a,b^**	41.81 ± 9.75	29.83 ± 10.15	28.50 ± 8.48	**0.008**
**Semantic fluency^a,b^**	23.30 ± 5.14	18.46 ± 3.78	15.43 ± 2.98	**<0.001**
**BNT**	14.73 ± 0.60	14.50 ± 0.15	14.07 ± 1.59	0.361
**Digit span forward**	5.44 ± 1.25	5.00 ± 0.89	5.41 ± 1.31	0.570
**Digit span backward**	4.07 ± 1.21	3.36 ± 0.67	3.33 ± 0.89	0.059

CU: cognitively unimpaired, sMCI: stable mild cognitive impairment, pMCI: progressive mild cognitive impairment, MMSE: Mini Mental State Examination, OVMPT: Oktem Verbal Memory Processes Test, IR: immediate recall, FR: free recall, TR: total recall, BNT: Boston Naming Test.

^a^Indicates difference between CU and pMCI.

^b^Indicates difference between CU and sMCI.

^c^Indicates difference between sMCI and pMCI.

*Note*: *p* < 0.05 considered as significant in one‐way ANOVA.^1^

^1^ Bold values indicate statistically significant results.

**TABLE 3:** Follow‐up neuropsychological test scores of participants.

	CU (*n* = 27)	sMCI (*n* = 13)	pMCI (*n* = 14)	*p*
**MMSE^a,b,c^**	29.31 ± 0.84	26.39 ± 2.79	23.21 ± 3.58	**<0.001**
**OVMPT‐total score^a,b,c^**	117.77 ± 13.49	83.46 ± 20.50	60.00 ± 15.65	**<0.001**
**OVMPT‐IR^a,b^**	5.77 ± 1.68	4.39 ± 1.26	3.50 ± 1.09	**<0.001**
**OVMPT‐FR^a,b,c^**	13.73 ± 1.56	6.85 ± 4.51	1.43 ± 2.85	**<0.001**
**OVMPT‐TR^a,c^**	14.96 ± 0.20	14.31 ± 0.95	10.43 ± 2.38	**<0.001**
**Semantic fluency^a^**	21.92 ± 5.01	18.08 ± 5.78	14.93 ± 5.12	**0.001**
**Lexical fluency^a,b^**	40.86 ± 15.62	25.58 ± 12.27	24.67 ± 13.31	**0.008**
**Stroop interference^b^**	44.44 ± 14.91	70.30 ± 27.71	51.50 ± 23.30	**0.006**
**BNT**	14.88 ± 0.33	14.15 ± 2.19	14.35 ± 0.84	0.151
**Digit span forward**	5.19 ± 1.05	5.00 ± 1.00	5.21 ± 1.37	0.858
**Digit span backward**	4.11 ± 1.07	3.08 ± 1.24	3.35 ± 1.39	**0.032**

CU: cognitively unimpaired, sMCI: stable mild cognitive impairment, pMCI: progressive mild cognitive impairment, MMSE: Mini Mental State Examination, OVMPT: Oktem Verbal Memory Processes Test, IR: immediate recall, FR: free recall, TR: total recall, BNT: Boston Naming Test.

^a^Indicates difference between CU and pMCI.

**^b^** Indicates difference between CU and sMCI.

^c^Indicates difference between sMCI and pMCI.

*Note*: *p* < 0.05 considered as significant in one‐way ANOVA.^2^

^2^ Bold values indicate statistically significant results.

### 2.3 EEG Recordings, Paradigm, and Analysis

All EEGs are recorded in a sound‐attenuated, electrically isolated and dimly lighted Faraday room in morning hours. EEG was recorded from 30 electrodes positioned on an elastic cap following the international 10–20 system. Reference electrodes were placed on the earlobes (A1–A2), and an electrooculogram was recorded from medial upper and lateral orbital location of the right eye. Electrode impedances were maintained below 10 kΩ, and EEG signals were digitized at a rate of 500 Hz using a BrainAmp 32‐channel DC amplifier with a band limit of 0.03–70 Hz. A classical visual oddball paradigm was employed, consisting of two types of stimuli: target and standard. Participants were presented with a total of 120 stimuli (40 target and 80 standard) on a 22″ screen with a refresh rate of 60 Hz. The duration of each stimulus was 1 s, and the interval between stimuli varied randomly between 3 and 7 s. The luminance of the target stimulus was 40 cd/m^2^, while the standard stimulus had a luminance of 10 cd/m^2^. Participants were instructed to mentally count the target stimuli and report the count at the end of the recording session.

Data preprocessing was conducted using Brain Vision Analyzer software from Brain Products GmbH, Germany. The raw EEG data underwent preprocessing steps including applying a high‐pass filter with a zero‐phase shift Butterworth at 0.1 Hz and notch filters at 50 Hz to remove line noise. Data was further filtered between 0.5 and 30 Hz. Baseline correction was applied in relation to 200 ms of pre‐stimulus. Trials containing target stimuli were segmented into epochs of 1000 ms duration, consisting of a 200 ms pre‐stimulus period and an 800 ms post‐stimulus period. The artifacts were removed using automatic artifact rejection based on specific criteria: (1) maximum amplitude within an epoch: ±50 µV; (2) maximum allowed voltage step: 50 µV/ms; (3) maximum allowed difference within a 200 ms interval: 50 µV; and (4) minimum activity within a 100 ms interval: 0.5 µV. There were no differences between groups in terms of epoch count in both baseline and follow‐up EEG recordings and there were no within group differences [*F*_(2,51)_ = 0.983, *p* = 0.381] in repeated measures ANOVA (Table 1). The remaining artifact‐free epochs were averaged to obtain P300. Mean P300 amplitudes was measured automatically in 250–550 ms from *F_z_*, *C_z_*, and *P_z_* electrodes.

### 2.4 Statistical Analysis

All statistical analysis was carried out with SPSS 29.0 package program. A mixed design ANOVA with between‐subject factor Group [3 levels: CU, sMCI, pMCI] and within‐subject factors anterior–posterior electrode locations (AP) [3 levels: *F_z_*, *C_z_*, *P_z_*] and time [2 levels: baseline, follow‐up] was conducted. Correlations between neuropsychological tests and amplitudes were investigated by using the Pearson correlation coefficient. The *p* value less than 0.05 was considered significant in ANOVAs. To account for multiple comparisons across correlation analyses, Bonferroni correction was applied, resulting in a corrected significance threshold of *p* < 0.0036. Greenhouse–Geisser values were reported. Post hoc comparisons were performed using Bonferroni corrections. The ROC curve analysis was applied to baseline P300 amplitude values. In order to determine cut‐off scores Youden indices were calculated.

### 2.5 Linear Mixed‐Effects Model (LMM) Analyses

Longitudinal changes in P300 mean amplitudes were examined using linear mixed‐effects models (LMMs). Separate models were constructed for each electrode site (*F_z_*, *C_z_*, and *P_z_*). Group (CU, sMCI, pMCI), Time (baseline, follow‐up), and the Group × Time interaction were included as fixed effects. A random intercept for participant was specified to account for within‐subject dependence arising from repeated measurements over time. To account for potential effects of demographic and baseline cognitive differences on P300 amplitudes, additional linear mixed‐effects models were conducted including age and baseline MMSE score as covariates. Group, Time, and Group × Time interaction terms were entered as fixed effects for each electrode site (*F_z_*, *C_z_*, and *P_z_*).

Models were estimated using restricted maximum likelihood (REML). Fixed effects were evaluated using Wald *χ* ^2^ tests. When significant main or interaction effects were detected, post hoc pairwise comparisons were conducted using estimated marginal means (EMMs) with Bonferroni correction for multiple comparisons. Estimated regression coefficients (*β*), standard errors (SE), and 95% confidence intervals (CIs) were reported where appropriate.

To quantify explained variance, marginal *R* ^2^ (variance explained by fixed effects) and conditional *R* ^2^ (variance explained by the full model including random effects) were calculated. Statistical significance was set at *p* < 0.05 (two‐tailed). All analyses were performed using linear mixed modeling procedures appropriate for unbalanced longitudinal data.

## 3 Results

### 3.1 Results of the LMM Analysis in P300 Mean Amplitudes

#### 3.1.1 P300 Mean Amplitude at *F_z_*

For *F_z_*, the LMM revealed a significant main effect of Group (*χ* ^2^(2) = 23.86, *p* < 0.001), indicating overall differences in frontal P300 amplitude across diagnostic groups. The main effect of Time was not significant (*χ* ^2^(1) = 2.14, *p* = 0.144), and the Group × Time interaction was also not significant (*χ* ^2^(2) = 0.59, *p* = 0.744).

At baseline, CU showed higher *F_z_* amplitudes than pMCI (Δ = 2.36 µV, *p* < 0.001), whereas differences between CU and sMCI and between sMCI and pMCI did not reach significance. At follow‐up, CU showed higher amplitudes than both sMCI (Δ = 1.25 µV, *p* = 0.032) and pMCI (Δ = 2.29 µV, *p* < 0.001), while sMCI and pMCI did not differ. Within‐group contrasts indicated a modest but significant decline over time in sMCI (*p* = 0.044), whereas CU and pMCI showed no significant longitudinal change.

#### 3.1.2 P300 Mean Amplitude at *C_z_*

A significant Group × Time interaction was observed (*χ* ^2^(2) = 10.78, *p* = 0.005), indicating that longitudinal changes in central P300 amplitude differed across groups.

Post hoc comparisons showed that at baseline, CU exhibited higher *C_z_* amplitudes than sMCI (Δ = 1.53 µV, *p* < 0.001) and pMCI (Δ = 3.00 µV, *p* < 0.001), and sMCI also showed higher amplitudes than pMCI (Δ = 1.47 µV, *p* = 0.002). At follow‐up, this graded pattern persisted, with CU remaining higher than sMCI and pMCI (both *p* < 0.001) and sMCI remaining higher than pMCI (*p* = 0.009).

Within‐group analyses revealed a significant longitudinal decline in *C_z_* amplitude in all groups, with the largest decrease observed in sMCI (Δ = 1.15 µV, *p* < 0.001), followed by pMCI (Δ = 0.90 µV, *p* < 0.001), and a smaller but significant decline in CU (Δ = 0.21 µV, *p* = 0.021).

At *C_z_*, the LMM demonstrated a robust main effect of Group (*χ* ^2^(2) = 30.54, *p* < 0.001) and a significant main effect of Time (*χ* ^2^(1) = 19.64, *p* < 0.001).

#### 3.1.3 P300 Mean Amplitude at *P_z_*

For *P_z_*, the LMM showed a significant main effect of Group (*χ* ^2^(2) = 34.62, *p* < 0.001) and a significant main effect of Time (*χ* ^2^(1) = 18.32, *p* < 0.001). The Group × Time interaction did not reach statistical significance.

At baseline, CU exhibited higher parietal P300 amplitudes than both sMCI (Δ = 1.21 µV, *p* = 0.014) and pMCI (Δ = 2.76 µV, *p* < 0.001), and sMCI amplitudes were higher than those of pMCI (Δ = 1.55 µV, *p* = 0.006). At follow‐up, CU continued to show higher amplitudes than sMCI (Δ = 2.02 µV, *p* < 0.001) and pMCI (Δ = 2.14 µV, *p* < 0.001), whereas sMCI and pMCI no longer differed.

Longitudinal contrasts indicated a significant decline in *P_z_* amplitude in CU (Δ = 0.63 µV, *p* < 0.001) and sMCI (Δ = 1.43 µV, *p* < 0.001), while pMCI showed no significant change over time (*p* = 0.945).

Additional covariate‐adjusted linear mixed‐effects analyses including age and baseline MMSE scores demonstrated that neither covariate showed significant effects on P300 amplitudes across electrode sites (all *p* > 0.05)

### 3.2 Results of the ANOVA Analysis in P300 Mean Amplitudes

The mixed‐design ANOVA showed a significant main GROUP effect [*F*_(2,51)_ = 16.346, *p* < 0.001] in P300 amplitude values indicating sMCI and pMCI had lower amplitudes compared to CU (*p* < 0.001, *p* = 0.001 respectively), with a large effect size (partial *η* ^2^ = 0.39). There was a main TIME effect [*F*_(1,51)_ = 12.026, *p* = 0.001] corresponding to a moderate‐to‐large effect size (partial *η* ^2^ = 0.19), showing baseline P300 amplitudes are higher in comparison to follow‐up regardless of the group. Additionally, there was a significant interaction effect [*F*_(2,51)_ = 3.325, *p* = 0.044] between GROUP and TIME with a small‐to‐moderate effect size (partial *η* ^2^ = 0.12). Post hoc analysis showed that the follow‐up P300 amplitudes of CU and sMCI were decreased in comparison to baseline amplitudes (*p* = 0.043, *p* < 0.001 respectively). It was shown that CU had higher P300 amplitudes compared to sMCI and pMCI (*p* = 0.036, *p* < 0.001 respectively), and sMCI had higher amplitudes compared to pMCI (*p* = 0.008) in baseline. In follow‐up, it was found that CU had higher P300 amplitudes compared to sMCI and pMCI (all, *p* < 0.001), however there was no significant difference between sMCI and pMCI groups.

Covariate‐adjusted analyses demonstrated that neither age nor baseline MMSE scores showed significant effects on P300 amplitudes (all *p* > 0.05). Importantly, the primary group‐related findings remained unchanged following adjustment for these variables.

### 3.3 Results of the ROC Analysis

ROC curve analyses demonstrated that P300 amplitude discriminated between diagnostic groups.

### 3.4 Baseline P300 Amplitudes in CU and pMCI

For cognitively unimpaired individuals versus pMCI, the *P_z_* (AUC = 0.915, *p* < 0.001) electrode showed a sensitivity of 92.9%, specificity of 81.5%, cut‐off amplitude of 2.30 mV, and a Youden index of 0.75. *C_z_* (AUC = 0.902, *p* < 0.001) yielded a sensitivity of 92.9%, specificity of 88.9%, and a Youden index of 0.82 at the cut‐off amplitude of 2.11 µV, while *F_z_* (AUC = 0.778, *p* = 0.004) showed a sensitivity of 85.7%, specificity of 63%, cut‐off amplitude of 2.30 µV.

### 3.5 Baseline P300 Amplitudes in CU and sMCI

In distinguishing cognitively unimpaired participants from sMCI, *C_z_* (AUC = 0.718, *p* = 0.027) reached a sensitivity of 100% with specificity of 40.7% at the cut‐off amplitude 4.33 µV, and *P_z_* (AUC = 0.749, *p* = 0.012) showed sensitivity of 53.8% with specificity of 88.9% with cut‐off amplitude of 2.10 µV.

### 3.6 Baseline P300 Amplitudes in sMCI and pMCI

For stable versus progressive MCI, both *C_z_* (AUC = 0.893, *p* = 0.001) and *P_z_* (AUC = 0.835, *p* = 0.001) demonstrated sensitivity of 78.6% and specificity of 92.3%, with cut‐off amplitudes of 1.39 µV and 1.20 µV, respectively, corresponding to Youden indices of 0.71. P300 amplitudes measured from *F_z_* (AUC = 0.736, *p* = 0.037) demonstrated sensitivity of 78.6% and specificity of 69.2%, at the cut‐off score of 3.09 µV.

The sensitivity, specificity values, cut‐off scores and Youden indices are presented in Table 4.

**TABLE 4:** Baseline results of the ROC curve analysis.

	Cognitively unimpaired vs. progressive MCI		
	*F_z_*	*C_z_*	*P_z_*
**Sensitivity (%)**	85.7	92.9	92.9
**Specificity (%)**	63	88.9	81.5
**Cut‐off scores (mV)**	3.10	2.11	2.30
**Youden index**	0.49	0.82	0.75
	**Cognitively unimpaired vs. stable MCI**		
**Sensitivity (%)**	NA	100	53.8
**Specificity (%)**		40.7	88.9
**Cut‐off scores (mV)**		4.33	2.10
**Youden index**		0.41	0.43
	**Stable MCI vs. progressive MCI**		
**Sensitivity (%)**	78.6	78.6	78.6
**Specificity (%)**	69.2	92.3	92.3
**Cut‐off scores (mV)**	3.09	1.39	1.20
**Youden index**	0.48	0.71	0.71

ROC: receiver operating characteristics, MCI: mild cognitive impairment, mV: microvolt.

### 3.7 Baseline Correlations

There were moderate significant correlations between baseline P300 amplitudes measured from *F_z_* and OVMPT‐Total score (*r* = 0.481, *p* < 0.001), OVMPT‐IR (*r* = 0.396, *p* = 0.003), OVMPT‐FR (*r* = 0.436, *p* = 0.001), OVMPT‐TR (*r* = 0.457, *p* = 0.001). There were moderate to strong significant correlations between P300 amplitudes measured from *C_z_* and MMSE (*r* = 0.493, *p* < 0.001), OVMPT‐Total score (*r* = 0.624, *p* < 0.001), OVMPT‐IR (*r* = 0.579, *p* < 0.001), OVMPT‐FR (*r* = 0.544, *p* < 0.001), OVMPT‐TR (*r* = 0.560, *p* < 0.001), and semantic fluency scores (*r* = 0.480, *p* < 0.001). P300 amplitudes from *P_z_* electrode location showed moderate to strong correlations with MMSE (*r* = 0.548, *p* < 0.001), OVMPT‐Total score (*r* = 0.643, *p* < 0.001), OVMPT‐IR (*r* = 0.611, *p* < 0.001), OVMPT‐FR (*r* = 0.567, *p* < 0.001), OVMPT‐TR (*r* = 0.445, *p* = 0.001), semantic fluency (*r* = 0.542, *p* < 0.001).

### 3.8 Follow‐Up Correlations

There were moderate significant correlations between follow‐up P300 amplitudes measured from *F_z_* and MMSE (*r* = 0.451, *p* = 0.001), OVMPT‐FR (*r* = 0.407, *p* = 0.002), lexical fluency (*r* = 0.547, *p* < 0.001), and digit span backward (*r* = 0.409, *p* = 0.0032). There were moderate to strong significant correlations between P300 amplitudes measured from *C_z_* and MMSE (*r* = 0.472, *p* < 0.001), OVMPT‐Total score (*r* = 0.520, *p* < 0.001), OVMPT‐FR (*r* = 0.521, *p* = 0.002), semantic fluency scores (*r* = 0.480, *p* < 0.001), lexical fluency (*r* = 0.526, *p* < 0.001), and digit span backward (*r* = 0.447, *p* = 0.001). P300 amplitudes from *P_z_* electrode location showed moderate to strong correlations with MMSE (*r* = 0.463, *p* < 0.001), OVMPT‐Total score (*r* = 0.510, *p* < 0.001), OVMPT‐FR (*r* = 0.414, *p* < 0.001), OVMPT‐TR (*r* = 0.420, *p* = 0.002), semantic fluency (*r* = 0.401, *p* = 0.0031), lexical fluency (*r* = 0.495, *p* = 0.001), and digit span backward (*r* = 0.438, *p* = 0.001).

## 4 Discussion

This study examined longitudinal changes in P300 event‐related potential amplitudes in cognitively unimpaired individuals and in stable and progressive mild cognitive impairment. Our findings strongly support P300 amplitude as a sensitive, non‐invasive neurophysiological marker of cognitive aging and decline, capturing both cross‐sectional group differences and within‐subject longitudinal trajectories.

### 4.1 P300 as a Marker of Neurodegeneration

Our longitudinal LMM analyses demonstrated reduced P300 amplitudes in MCI, particularly in pMCI, along with significant central (*C_z_*) and parietal (*P_z_*) declines over time. These findings align with accumulating evidence that P300 amplitude is sensitive to early neurodegenerative processes. Reduced amplitudes and prolonged latencies have consistently been reported in MCI and Alzheimer's disease, reflecting impaired attentional allocation, working‐memory updating, and cognitive processing efficiency (Polich 2007; Bennys et al. 2007; Tarawneh and Holtzman 2021; Demirayak et al. 2023).

Meta‐analytic evidence further indicates that midline P300 amplitudes (*C_z_*, *P_z_*) are significantly smaller in AD compared to healthy controls, supporting its role as a robust group‐level neural marker (Hedges et al. 2016). Importantly, amplitude reductions are associated with deficits in attention and working memory, core domains affected in prodromal AD, and correlate with neuropsychological performance (Demirayak et al. 2023; Mohamed et al. 2024).

### 4.2 Differentiating sMCI and pMCI

At baseline, both MCI groups showed reduced amplitudes relative to CU, with the most pronounced reductions in pMCI (CU > sMCI > pMCI). This gradient likely reflects progressive neurophysiological disruption corresponding to clinical severity and future cognitive trajectory.

Critically, baseline P300 amplitudes distinguished sMCI from pMCI, whereas traditional neuropsychological tests did not. This suggests that electrophysiological measures can detect subtle neural dysfunction before overt behavioral decline. Similar findings have been reported in converter studies, where MCI individuals who progressed to AD showed lower baseline P300 amplitudes compared to non‐converters (Olichney et al. 2002; Bennys et al. 2007; Liang et al. 2025).

The intermediate profile of sMCI may reflect heterogeneous neural trajectories, potentially indicating early pathological disruption before clinical conversion. Prior literature suggests that P300 abnormalities may precede overt cognitive symptoms and serve as a prognostic biomarker of progression risk (Cintra et al. 2015).

### 4.3 Longitudinal Trajectories

A significant main effect of time showed that P300 amplitude declined across groups. In CU participants, this reduction was modest and likely reflects normal age‐related cortical slowing and reduced synaptic efficiency (Ishii et al. 2017; Pascarella et al. 2025). In contrast, sMCI exhibited more pronounced decline, suggesting heightened neural vulnerability despite absence of dementia diagnosis.

Interestingly, the pMCI group did not show significant longitudinal amplitude reduction. This may reflect a floor effect or a neurodegenerative plateau, where cortical dysfunction has reached a critical threshold. Similar plateau effects have been described in structural imaging studies of converters (Jack et al. 2010; Dickerson and Wolk 2012; Verovnik et al. 2023). These findings suggest that P300 amplitude reflects not only current neural efficiency but also remaining capacity for adaptation or further decline.

The significant Group × Time interaction supports the presence of distinct electrophysiological trajectories across CU, sMCI, and pMCI. This pattern aligns with biomarker cascade models positioning electrophysiological changes early in AD pathophysiology (Jack et al. 2018; Hampel et al. 2018).

### 4.4 Discriminative Accuracy (ROC Findings)

ROC analyses demonstrated strong diagnostic performance of P300 amplitude. *C_z_* and *P_z_* amplitudes differentiated CU from pMCI with AUC values exceeding 0.90, indicating excellent discriminative power. These findings are consistent with previous reports emphasizing centroparietal P300 accuracy in early AD detection (Polich and Corey‐Bloom 2005; Porcaro et al. 2019; Gonzalez‐Montealegre et al. 2025).

Moderate AUC values for CU vs. sMCI (∼0.72–0.75) likely reflect heterogeneity within sMCI, including variability in pathology and compensatory mechanisms (Mattay et al. 2006; Petersen et al. 2018). Nevertheless, even this level of discrimination highlights the sensitivity of P300 to early vulnerability.

Importantly, baseline P300 amplitudes also differentiated sMCI from pMCI with high accuracy (*C_z_*: 0.893; *P_z_*: 0.835), reinforcing its prognostic potential and supporting multimodal risk stratification frameworks (Albert et al. 2011; Dubois et al. 2016).

The superior diagnostic accuracy at *C_z_* can be explained neurophysiologically. P300 reflects coordinated activation of parietal–temporal association cortices, posterior cingulate, and prefrontal regions. Bilateral dipoles converge at the midline and project maximally to central scalp locations (Linden 2005; Polich 2007). Because *C_z_* captures integration of frontal and parietal sources, it indexes attentional resource allocation and working‐memory updating which are the domains especially vulnerable in early AD (O'Connell et al. 2012).

Studies have shown that *C_z_*‐recorded ERPs predict conversion from MCI to AD (Chapman et al. 2011) and yield higher sensitivity and specificity than parietal sites (Polikar et al. 2010; Mohamed et al. 2024). Additionally, aging studies demonstrate an anterior shift of P3b activity from parietal to central regions, reflecting posterior atrophy and compensatory frontal recruitment (Fjell and Walhovd 2010; Li et al. 2023). Thus, the central midline region may remain particularly sensitive to early neurodegenerative change.

### 4.5 Relationship With Cognitive Performance

Correlational analyses showed meaningful associations between P300 amplitude and cognitive function. Baseline *F_z_* and *C_z_* amplitudes correlated with MMSE and episodic memory, suggesting involvement of frontal–central networks in encoding and retrieval (Yener and Başar 2010; Chapman et al. 2011).

*P_z_* amplitudes were associated with semantic fluency, lexical fluency, and digit span, reflecting parietal contributions to executive–linguistic integration (Polich 2007; Gazzaley and D'Esposito 2007). Associations with digit span backward further support involvement of the fronto‐parietal control network, known to deteriorate early in MCI (Belleville et al. 2007; Luck 2021). Importantly, these relationships persisted at follow‐up, demonstrating longitudinal stability and functional relevance.

### 4.6 Clinical Implications

Together, these findings indicate that P300 amplitude reflects both current cognitive state and future decline risk. Unlike neuropsychological tests which are influenced by cognitive reserve and compensatory mechanisms (Stern 2012), ERPs measure neural efficiency with millisecond precision (Polich 2007). Reduced P300 amplitude likely reflects synaptic inefficiency and cortical slowing in temporo‐parietal and cingulate networks affected early in AD (Linden 2005; Fjell and Walhovd 2010).

P300 recording is low‐cost, non‐invasive, and widely accessible, making it suitable for clinical screening, longitudinal monitoring, and early‐stage clinical trials. It may be particularly valuable in settings where molecular or imaging biomarkers are limited.

### 4.7 Limitations and Future Directions

The relatively small sample size limits generalizability. Additionally, absence of biomarker‐confirmed pathology restricts definitive classification of underlying AD processes. Future studies should incorporate multimodal validation to determine specificity of P300 changes.

Longer follow‐up periods are needed to determine whether early P300 alterations in sMCI predict eventual AD conversion. Intervention studies could examine whether pharmacological or behavioral treatments modulate P300 dynamics, potentially establishing it as a treatment‐response biomarker.

## 5 Conclusion

In conclusion, P300 amplitude reflects early neural dysfunction and cognitive trajectory in aging and MCI. Cross‐sectional and longitudinal differences, strong diagnostic accuracy, and meaningful cognitive correlations collectively support its role as a promising non‐invasive electrophysiological marker for identifying individuals at elevated risk for Alzheimer's disease.

## Author Contributions

**Yağmur Özbek**: conceptualization, methodology, data collection, analysis, writing – original draft. **Görsev G. Yener**: supervision, clinical diagnosis, methodology, writing – review & editing. All authors approved the final manuscript.

## Funding

The authors have nothing to report.

## Ethics Statement

The authors confirm that this manuscript is original, not under consideration elsewhere, and complies with ICMJE authorship criteria and COPE ethical guidelines. All research procedures involving human participants adhered to institutional and international ethical standards. This study was conducted in accordance with the Declaration of Helsinki. Ethical approval was obtained from the appropriate Institutional Review Board prior to participant recruitment.

## Consent

Written informed consent was obtained from all participants or their legally authorized representatives.

## Conflicts of Interest

The authors declare that they have no competing financial interests or personal relationships that could have influenced the work reported in this paper.

## Permission to Reproduce Material

No previously published material requiring permission has been reproduced in this manuscript.

## Clinical Trial Registration

This study was observational and was not registered as a clinical trial.

## Data Availability

The data that support the findings of this study are available from the corresponding author upon reasonable request, subject to institutional ethics approval and data protection regulations. Due to the sensitive nature of clinical and neurophysiological data, publicly sharing individual‐level data is restricted.
